# Boosting-Based On-Road Obstacle Sensing Using Discriminative Weak Classifiers

**DOI:** 10.3390/s110404372

**Published:** 2011-04-14

**Authors:** Shyam Prasad Adhikari, Hyeon-Joong Yoo, Hyongsuk Kim

**Affiliations:** 1 Division of Electronics and Information Engineering, Chonbuk National University, Jeonju 561-756, Korea; E-Mails: shyam.rvision@hotmail.com (S.P.A.); hskim@jbnu.ac.kr (H.K.); 2 Department of IT Engineering, Sangmyung University, Chonan 330-720, Korea

**Keywords:** weak classifiers, Haar-like features, AdaBoost, quadratic discriminant analysis

## Abstract

This paper proposes an extension of the weak classifiers derived from the Haar-like features for their use in the Viola-Jones object detection system. These weak classifiers differ from the traditional single threshold ones, in that no specific threshold is needed and these classifiers give a more general solution to the non-trivial task of finding thresholds for the Haar-like features. The proposed quadratic discriminant analysis based extension prominently improves the ability of the weak classifiers to discriminate objects and non-objects. The proposed weak classifiers were evaluated by boosting a single stage classifier to detect rear of car. The experiments demonstrate that the object detector based on the proposed weak classifiers yields higher classification performance with less number of weak classifiers than the detector built with traditional single threshold weak classifiers.

## 1. Introduction

**Figure 1.**
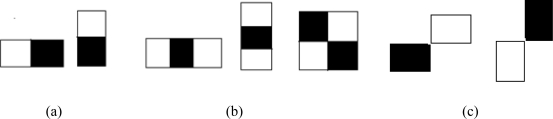
Examples of the Haar-like feature set. (**a**) Basic feature set which consists of two adjacent rectangles. (**b**) and (**c**) Extended feature sets which consist of different number and arrangement of rectangles, respectively.

In pattern recognition, object detection generally is a two-class classification problem with two essential issues of feature selection and classifier design based on the selected features. Classifiers based on Haar-like features [1] have been successfully used for object detection. Viola and Jones [2] proposed an object detection framework where these Haar-like features are selected and classifier is trained using AdaBoost [3]. This approach has become a popular framework for object detection and several extensions of this framework have been proposed. One of the extensions is the improvement in the boosting algorithm. Modified versions of AdaBoost such as Real AdaBoost [4], FloatBoost [5] and KLBoosting [6] are available. Real AdaBoost is used for multi-view face detection [7]. In addition to face detection [5] Float Boost is also applied to hand shape detection [8]. The other extension of the original framework is to use an extended set of Haar-like features so that different image patterns can be evaluated. In addition to the basic feature set of Figure 1(a), an extended set of Haar-like features as shown in Figure 1(b,c) are introduced in [9,10], and [11]. Mita *et al*. [12] have selected multiple co-occurring linear weak classifiers to form a more efficient classifier. Boosting in a hierarchical feature space where the local Haar-like features are replaced by global features derived from PCA in later stages of boosting is introduced in [13]. An extension of Haar-like features in which different weights, determined by techniques like Brute force search, Genetic algorithms and Fischer’s linear discriminant analysis, are assigned to the rectangles of Haar-like features is proposed in [14]. Hybrid features composed of gradient features, Edgelet features and Haar-like features are used in [15] for pedestrian detection.

The selection of threshold for the Haar-like features is not a trivial task and has not been explained in detail in [2]. The weak classifiers based on single threshold Haar-like features are sub-optimal and not efficient for discriminating object and non-object. At later stages of the cascade these single threshold Haar-like features become too weak for discrimination and make boosting ineffective [13]. In this paper, we propose a different set of weak classifiers for boosting that achieves higher classification accuracy with less number of weak classifiers. Unlike in [2], the proposed weak classifiers do not require explicit thresholds be calculated for the Haar-like features and present a more general solution to the threshold selection problem. The proposed weak classifiers are equally efficient for discrimination at later stages of boosting also.

The rest of the paper is organized as follows: Section 2 describes the AdaBoost learning of the Haar-like features. Section 3 presents the proposed method for realizing efficient weak classifiers. Experimental setup and results are presented in Section 4, followed by concluding remarks in Section 5.

## 2. Boosting of Weak Classifiers

This section describes the conventional weak classifiers and AdaBoost learning algorithm for constructing a strong classifier by selecting the weak classifiers.

### 2.1. Boosting of Weak Classifiers

**Figure 2.**
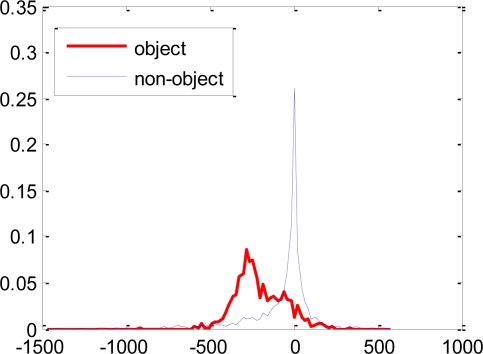
Example of feature value distributions. In [2] a single threshold that separates the two distributions is used.

The Haar-like features have scalar values that represent the difference in the sum of intensities between the adjacent rectangular regions. To capture the *ad hoc* knowledge about the domain, these features are evaluated at different positions and with different sizes exhaustively according to the base resolution of the classifier. For example, when the classifier resolution is 24 × 18 pixels, 91,620 features are generated from the five features in Figure 1(a,b). Each feature is evaluated on all the training samples and the probability density for each of the object and non-object class is calculated as shown in Figure 2. In [2], a single threshold that separates these two distributions is selected for each feature. These features along with their respective thresholds and polarity form the weak classifiers for the learning algorithm.

A weak classifier can be mathematically described as:

(1) $$h(x,f,p,\theta )=\{\begin{array}{c} 1\text{ if }\mathrm{pf}(x)>p\theta \\ -1\text{ otherwise } \end{array}$$

where *x* is the base resolution of the classifier, *f* the Haar-like feature, *θ* the threshold for the feature and *p* the polarity indicating the direction of inequality. The choice of optimal threshold for the features is not stated clearly in [2] and shows to be a non-trivial task.

### 2.2. AdaBoost

AdaBoost is a machine learning boosting algorithm that constructs a strong classifier by combining a set of weak classifiers. A small number of discriminative weak classifiers are selected by updating the sample distribution. The prediction of the strong classifier is produced through a weighted majority voting of the weak classifiers. Pseudo code of a variant of AdaBoost used in the implementation is given in Algorithm 1.

## **Algorithm 1.** Pseudo code of Discrete AdaBoost

-Given example images (*x*_1_, *y*_1_),..., (*x_n_*, *y_n_*) where *y_i_* = −1,1 for negative and positive examples.
-Initialize weights $w_{1,i}=\frac{1}{l+m}\;\;$, where *l* and *m* are the number of positives and negatives respectively
-For *t =* 1,...,*T*:
  1. Normalize the weights,
    $$w_{t,i}\leftarrow \frac{w_{t,i}}{\sum_{j=1}^{n}w_{t,i}}$$
  2. Select the best weak classifier with respect to the weighted error:
    $${ɛ}_{t}={\text{min}}_{f,p,\theta }\sum_{i}w_{i}\left|h\left(x_{i},f,p,\theta \right)-y_{i}\right|$$
  3. Define *h_t_*(*x*) = *h*(*x*, *f_t_*, *p_t_*, *θ_t_*) where *f_t_*, *p_t_* and *θ_t_* are minimizers of *ɛ*_t_
  4. Update the weights: $w_{t+1,i}=w_{t,i}\{\begin{array}{lll} e^{-\alpha_{t}} & \text{if} & y_{i}=h_{t}(x_{i})\\ e^{\alpha_{t}} & \text{if} & y_{i}\neq h_{t}(x_{i}) \end{array}$where,
    $$\alpha_{t}=\frac{1}{2}\text{ln}\left(\frac{1-{ɛ}_{t}}{{ɛ}_{t}}\right)$$
– The final strong classifier is:
  $$H_{\mathrm{final}}(x)=\mathrm{sign}\left(\sum_{t=1}^{T}\alpha_{t}h_{t}(x)\right)$$

## 3. Proposed Weak Classifiers

This section describes the proposed weak classifiers which eliminate the need of explicit threshold for the Haar-like features. First we formulate the definition of the new weak classifiers based on Bayesian decision theory and quadratic discriminant analysis [16]. Later we discuss the motivation to use and the relative advantage of the proposed weak classifiers over the traditional single threshold weak classifiers.

### 3.1. Bayesian Decision Rule

Given a set of features, the Bayesian decision theory for classification requires decision boundaries that minimize the error rate on the training data. Let us consider a two class problem with *ω*_1_ and *ω*_2_ as the state of nature. If *x* is the observed feature value, the decision boundary that minimizes the classification error is given in terms of the posterior probabilities as *P*(*ω*_1_|*x*) = *P*(*ω*_2_|*x*). The corresponding decision rule is: decide *ω*_1_ if *P*(*ω*_1_|*x*) > *P*(*ω*_2_|*x*); else decide *ω*_2_.

### 3.2. Discriminant Function for Normal Density

One of the most useful ways to represent pattern classifiers is in terms of a set of discriminant functions *g_i_*(*x*); *i* = 1, 2,…, *c*, where *c* is the number of categories to discriminate. The classifier is said to assign a feature *x* to class *ω_i_* if:

(2) $$g_{i}(x)>g_{j}(x)\text{ for all }j\neq i$$

The effect of the decision rule is to divide the feature space into *c* decision regions. The regions are separated by decision boundaries, surfaces in the feature space where ties occur among the largest discriminant functions [16]. Assuming the distribution of the univariate Haar-like features to be normal, *i.e.*, *p*(*x*|*ω_i_*) ∼ *N*(*μ_i_*, Σ*_i_*), the minimum error rate classification can be achieved by the use of discriminant function of the form given in Equation (3) [16]:

(3) $$g_{i}(x)=-\frac{1}{2}{\left(x-\mu_{i}\right)}^{t}\Sigma_{i}^{-1}\left(x-\mu_{i}\right)-\frac{1}{2}\text{ln}2\pi -\frac{1}{2}\text{ln}\left|\Sigma_{i}\right|+\text{ln}P\left(\omega_{i}\right)$$

where *P*(*ω_i_*) is the priori probability of class *ω_i_*. Taking a general univariate normal case with different variances for each category, the resulting discriminant function is given as:

(4) $$g_{i}(x)=x^{t}W_{i}x+W_{i}x+w_{i0}$$

where:

$$\begin{array}{l} W_{i}=-\frac{1}{2}\Sigma_{i}^{-1}\\ W_{i}=\Sigma_{i}^{-1}\mu_{i}\\ w_{i0}=-\frac{1}{2}\mu_{i}^{t}\Sigma_{i}^{-1}\mu_{i}-\frac{1}{2}\text{ln}\left|\Sigma_{i}\right|+\text{ln}P\left(\omega_{i}\right) \end{array}$$

**Figure 3.**
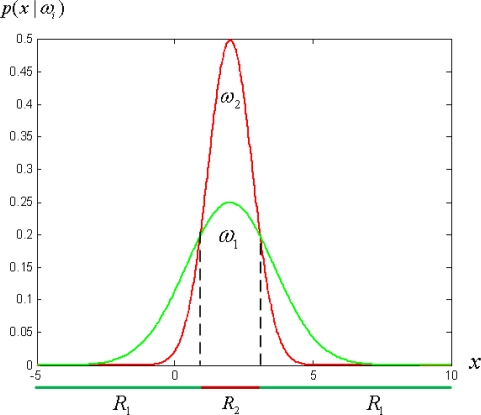
Non-simply connected decision regions in one dimension.

The discriminant functions of Equation (4) are inherently quadratic. The decision surfaces are hyperquadrics and in one dimensional case the decision regions needn’t be simply connected as shown in Figure 3. This observation motivates us to formulate new kind of weak classifiers without explicitly specifying the threshold for each weak classifier.

### 3.3. Proposed Weak Classifiers

The proposed weak classifiers are based on the quadratic discriminant functions described above. Each Haar-like feature from the pool of 91,620 features is evaluated on the training samples and one-dimensional probability densities for object and non-object classes are calculated. Assuming the density of each feature to be normal, the distributions of feature on the object and non-object classes are parameterized by their maximum likelihood estimators, *i.e*., mean *μ* and variance Σ. The distribution for the object (positive) class is *p*(*x*|*ω_p_*) ∼ *N*(*μ_p_*, Σ*_p_*), and for non-object (negative) class is *p*(*x*|*ω_n_*) ∼ *N*(*μ_n_*, Σ*_n_*). The decision regions for the two distributions are given from Equation (2), *i.e*., assign the observed feature value *x* to class *ω_p_* if:

(5) $$g_{p}(x)>g_{n}(x)$$

This decision rule divides the feature space into decision regions which needn’t be simply connected for the same class. The proposed weak classifiers for the Haar-like features are defined as:

(6) $$h\left(x,f,\mu_{p},\mu_{n},\Sigma_{p},\Sigma_{n}\right)=\{\begin{array}{l} 1\;\;\;\text{if }\;g_{p}(f(x))>g_{n}(f(x))\\ -1\text{ otherwise} \end{array}$$

where *x* is the base resolution of the classifier and *f* is the Haar-like feature. Since a more general model of the distribution is considered, the proposed weak classifiers are expected to perform better than the single threshold weak classifier.

For the weak classifiers of Equation (1), each feature produces a single scalar value and the decision boundary corresponds to a scalar threshold. But the choice of this threshold is not stated clearly in [2] and determination of an optimal threshold is a nontrivial task. The proposed weak classifiers of Equation (6) are more general and do not require any explicit representation of the threshold. In fact, the weak classifiers of Equation (1) are a special case of the proposed weak classifiers when Σ*_p_* and Σ*_n_* are identical. The weak classifiers based on single threshold commonly employ “average of means” of the two distributions, *i.e.*, (μ_p_ + μ_n_)/2, as decision threshold. Under this hypothesis, it is statistically observed that most of the Haar-like features are non-discriminative and inefficient for boosting.

**Figure 4.**
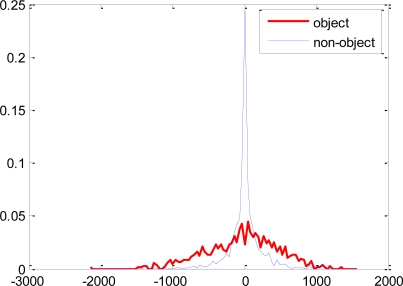
Typical distribution of feature values in later stages of boosting on the training data (described later).

The error rates of these single threshold weak classifiers selected at later stages of the boosting process become large as the sample distribution consists of samples which are difficult to discriminate as shown in Figure 4. The single threshold weak classifiers are not efficient in discriminating such distributions. The proposed weak classifiers are expected to efficiently discriminate the underlying distribution of Figure 4, as disjoint decision regions are also supported as shown in Figure 3.

## 4. Experimental Results

### 4.1. Data Preparation

**Figure 5.**
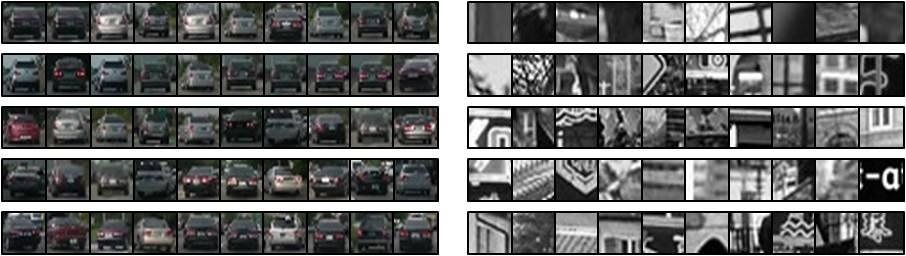
Example of the rear-of-car images (**left**) and the non-car images (**right**) used for training.

The experiments were carried out for detection of rear of cars. The experiments were done using 1,500 positive and 3,500 negative samples. The positive samples consisted of instances of rear of cars cropped from a video taken from a camera mounted at the front of a host car while driving in an urban environment. Each instance was resized to a base size of 24 × 18 pixels. The negative samples consisted of images cropped from random high resolution images that did not contain any instance of rear of car. Each negative sample was also resized to base size of 24 × 18 pixels. 1,000 positives and 3,000 negative samples were used for training the classifiers while the remaining 500 positive and 500 negative samples were used for validation. Figure 5 shows some of the positive and negative samples used for the experiment.

### 4.2. Performance Comparison between Proposed Weak Classifiers and Single Threshold Weak Classifiers

**Figure 6.**
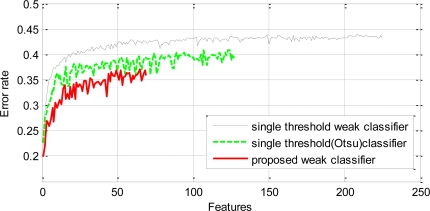
Error rates of the weak classifiers selected by boosting using proposed weak classifiers and the single threshold weak classifiers.

A single stage classifier was trained by AdaBoost on the training data using the proposed weak classifiers to achieve 100% hit rate on the positive samples and zero false positive on the negative samples. The final strong classifier achieved the required performance on the training data with a total of 69 proposed weak classifiers. The first weak classifier selected by AdaBoost yielded an error rate of 0.2. The subsequent selected weak classifiers yielded comparatively higher error rates. The worst error rate among the selected classifiers was 0.37 for the 66th classifier. The error rates of subsequent selected classifiers can be seen in Figure 6. Another strong classifier was trained on the same training data using the conventional single threshold based weak classifiers. These classifiers employed the average of means as the threshold. The final strong classifier required 225 weak classifiers to achieve similar performance on the training data. The error rate of the first selected weak classifier was 0.21 but it increased rapidly for the subsequent classifiers and the worst was 0.44 for the 208th classifier. A third strong classifier was trained on the same training data using single threshold based weak classifiers which employed Otsu’s method [17] for optimal threshold selection. The final classifier consisted of 125 weak classifiers. The error rate of the first weak classifier was 0.22 and the highest error rate was 0.41 for the 124th classifier. Figure 6 shows that the error rates of the proposed weak classifiers are consistently lower than the single threshold based counterparts.

**Figure 7.**
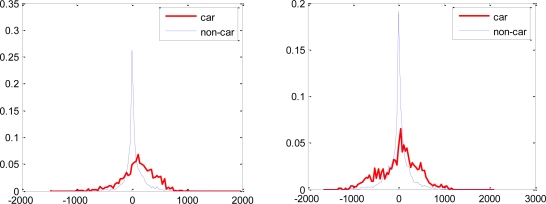
Distributions of feature values of the 6th (**left**) and the 9th (**right**) features selected by AdaBoost using the proposed weak classifiers. The features have error rate of 0.27 and 0.3 respectively under the proposed hypothesis. The single threshold approach will reject these features as inefficient since a single threshold is not sufficient to discriminate these types of distributions which are unimodal or close to unimodal.

Most of the features selected using the proposed weak classifiers have overlapping distributions of the object and non-object classes. Though these features have lower error rates and are boostable under the proposed hypothesis, they would have been rendered useless for boosting under the single threshold hypothesis. Some of the feature distributions are shown in Figure 7.

**Figure 8.**
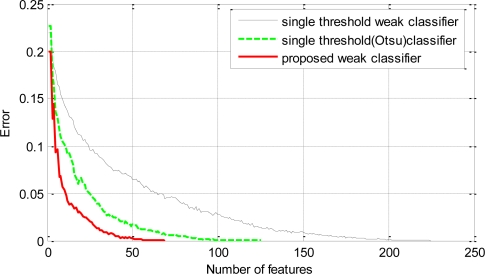
Plot of training error and the number of weak classifiers: the proposed weak classifiers and the single threshold (derived from average of means and Otsu’s optimal thresholding method) based weak classifiers.

The selection of efficient classifiers at each round of boosting helps the learning algorithm to converge faster (in terms of the number of weak classifiers) on the training data as can be seen in Figure 8. Similar performance (in terms of hit rate and false positive rate) on the training data can be achieved with a significant reduction in the total number of weak classifiers by using the proposed weak classifiers over the conventional single threshold weak classifiers.

**Figure 9.**
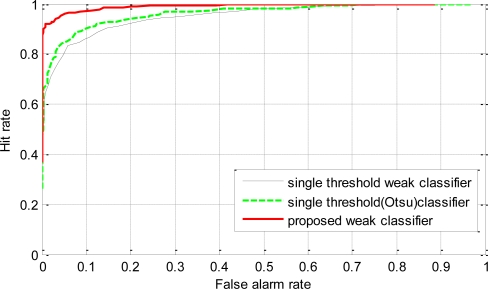
ROC curves for one stage classifiers trained using the proposed weak classifiers and single threshold (derived from average of means and Otsu’s optimal thresholding method) based weak classifiers.

To investigate the generalization performance of the proposed weak classifiers, the strong classifiers were tested on a validation dataset. The validation data set consisted of 500 positive and 500 negative sample images that were not used for training. ROC curves were generated using the validation dataset. The points in the ROC curves were obtained by evaluating each of the strong classifiers against the validation dataset by sliding the stage threshold from −10 to +10 at step of 0.25. The thresholds for the stage were chosen because varying the thresholds in this range proved to be sufficient to generate the whole range in the ROC curves. The plot of the hit rate *versus* the false alarm rate for all the methods is given in Figure 9. From the ROC curves in Figure 9, we can see that the classifier trained using the proposed weak classifiers perform consistently better than the classifier trained using single threshold weak classifiers. The detection rate of the classifier based on the proposed weak classifiers is always higher than the classifier based on single threshold weak classifiers. And for a given detection rate, the classifier using the proposed weak classifier always has less false alarm rate than the classifiers using single threshold weak classifiers. The higher performance of the proposed method reflects the benefit of the usage of discriminant function based weak classifiers, which are more effective at discriminating car and non-car examples.

### 4.3. Performance Comparison on Relatively Difficult Samples

**Figure 10.**
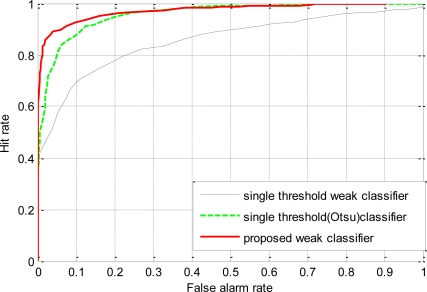
ROC curves for single stage classifier trained on difficult samples using the proposed weak classifiers and the single threshold weak classifiers. Single stage classifiers were trained on the negative samples acquired as false positives of an already trained 10 stage cascade using the single threshold based weak classifiers.

In this experiment, the classifiers were trained on relatively difficult samples than those of Section 4.2. The positive samples contained 500 positive images from the original training set. The negative samples contained 2,000 negative images. The negative samples were the false positives generated when a 10 stage cascade was evaluated on random high resolution images. The 10 stage cascade was trained on the original training set using the single threshold weak classifiers. In this sense, the negative samples are relatively difficult for the single threshold weak classifiers to discriminate. Three different classifiers were trained using the three types of weak classifiers on this data to achieve 100% hit rate and zero false positives. The classifier using the proposed weak classifiers required only 36 features whereas the classifier using single threshold weak classifier employing average of means required 236 features and the classifier employing Otsu’s thresholding method required 90 features to achieve same performance on the training data. This shows that the proposed weak classifiers are equally efficient in discriminating difficult samples than the single threshold counterparts. The generalization performance of the trained classifiers was tested on the validation set as in Section 4.2. The ROC curves in Figure 10, generated against the validation dataset show significant performance improvement of the classifier trained using the proposed weak classifiers over the classifier using single threshold weak classifiers.

### 4.4. Comparison of the Speed of the Object Detector

The speed of a cascaded object detector is directly proportional to the number of features evaluated per scanned sub-window in the image. To compare the speed of the detector, we trained three 15 stage cascade using the single threshold classifiers and the proposed weak classifiers on the UIUC Image database for car detection. The cascades were evaluated on the UIUC car test image set at different scales. For the classifier with single threshold employing average of means, an average of 14.5 features out of the total 248 features and for the one employing Otsu’s optimal threshold an average of 13 features out of the total of 230 were evaluated per sub-window, whereas for the proposed classifiers only an average of 8 features out of the total 131 features were evaluated per sub-window. Table 1 shows the total number of sub-windows scanned and the total features evaluated for seven images randomly sampled from the UIUC car test images at different scales. The feature value calculation time for the proposed classifier is the same as that for the single threshold Haar-like features. But from Equation (3) we see that the proposed weak classifier requires additional multiplication and addition operation to make the class decision. This makes them relatively more expensive to compute than the single threshold classifiers. The experiments conducted show that the proposed weak classifier requires around 1.6 times more computation time than the single threshold classifier to make a class decision. However as seen from Table 1, the single threshold classifiers need to evaluate on average around 1.6 times more features per sub-window than the proposed weak classifiers. This makes the speed of the proposed detector comparable to that of the conventional single threshold based detector.

## 5. Conclusions

In this paper, we have proposed a new set of weak classifiers for efficient boosting. The proposed weak classifiers do not require an explicit decision threshold to be calculated as is required for the single threshold weak classifiers and present a general solution for the optimal threshold finding problem. The proposed quadratic discriminant analysis based solution significantly improves the ability of the weak classifiers to discriminate object and non-object classes. The experimental results demonstrate that the proposed weak classifiers have far less classification error rate than the single threshold weak classifiers. An object detector trained using the proposed weak classifiers using AdaBoost facilitated efficient boosting and the final classifier yielded higher classification performance with less number of weak classifiers than a detector built with traditional single threshold weak classifiers.

## Figures and Tables

**Table 1.** Comparison of the speed of the detectors in terms of the average number of features evaluated per scanned window in the test images.

**S.No.**	**Total sub-windows scanned**	**Proposed Method**		**Single Threshold Method**			
				**Average of Means**		**Otsu’s Threshold**	

		**Total features evaluated**	**Average feature/sub-window**	**Total features evaluated**	**Average feature/sub-window**	**Total features evaluated**	**Average feature/sub-window**

1	63,313	521,545	8.23	868,037	13.7	790,047	12.4
2	63,618	541,644	8.51	929,962	14.61	859,328	13.5
3	85,106	692,548	8.13	1,173,507	14.9	1,032,627	12.13
4	87,378	763,826	8.74	1,302,362	14.9	1,122,984	12.85
5	40,810	366,326	8.97	653,903	16.3	620,030	15.19
6	82,354	688,846	8.36	1,078,020	13.1	1,052,336	12.77
7	58,590	492,783	8.41	842,548	14.38	753,639	12.86

## References

[1] Papageorgiou CP, Oren M, Poggio T A general framework for object detection.

[2] Viola P, Jones M Rapid object detection using a boosted cascade of simple features.

[3] Freund Y, Schapire R (1997). A decision-theoretic generalization of on-line learning and an application to boosting. J. Comput. Syst. Sci.

[4] Schapire RE, Singer Y (1999). Improved boosting algorithms using confidence-rated predictions. Mach. Learn.

[5] Liu C, Shum HY Kullback-leibler boosting.

[6] Li SZ, Zhang ZQ (2004). Floatboost learning and statistical face detection. IEEE Trans. Pattern Anal. Mach. Intell.

[7] Wu B, Ai H, Huang C, Lao S Fast rotation invariant multi-view face detection based on real adaboost.

[8] Ong EJ, Bowden R A boosted classifier tree for hand shape detection.

[9] Lienhart R, Maydt J (2003). An extended set of Haar-like features for rapid object detection. Proc. IEEE Int. Conf. Image Process.

[10] Wu B, Ai H, Huang C, Lao S Fast rotation invariant multi-view face detection based on real AdaBoost.

[11] Kolsch M, Turk M Robust hand detection.

[12] Mita T, Kaneko T, Stenger B, Hori H (2008). Discriminative feature co-occurrence selection for object detection. IEEE Trans. Pattern Anal. Mach. Intell.

[13] Zhang D, Li SZ, Perez DG Real-time face detection using boosting in hierarchical feature Spaces.

[14] Pavani SK, Delgado-Gomez D, Frangi AF (2010). Haar-like features with optimally weighted rectangles for rapid object detection. Pattern Recog.

[15] Hu B, Wang S, Ding X (2010). Multi features combination for pedestrian detection. J. Multimed.

[16] Duda R, Hart P, Stork D (2000). Pattern Classification.

[17] Otsu NA (1972). Threshold selection method from gray-level histograms. IEEE Trans. Syst. Man Cybern.

